# When Planning Screw Fracture Fixation Why the 5.5 mm Screw is the Goldilocks Screw: An Observational Computer Tomographic Study of Fifth Metatarsal Bone Anatomy in a Sample of Patients: Erratum

**DOI:** 10.1097/01.md.0000466623.90806.ef

**Published:** 2016-06-19

In the article “When Planning Screw Fracture Fixation Why the 5.5 mm Screw is the Goldilocks Screw: An Observational Computer Tomographic Study of Fifth Metatarsal Bone Anatomy in a Sample of Patients”,^1^ which appeared in Volume 94, Issue 18 of *Medicine*, Table 3 was presented incorrectly. The table has since been corrected and renamed as Figure 1, and the article has been corrected online.
